# Syrian females with congenital adrenal hyperplasia: a case series

**DOI:** 10.1186/s13256-022-03609-y

**Published:** 2022-10-15

**Authors:** Nada Dehneh, Rami Jarjour, Sahar Idelbi, Assad Alibrahem, Sahar Al Fahoum

**Affiliations:** 1 Dept. of Biochemistry and Microbiology, Faculty of Pharmacy, Damascus University, Damascus, Syria; 2 Clinical Genetics Unit, Atomic Energy Commission of Syria (AECS), Damascus, Syria; 3 Faculty of Pharmacy, Arab International University (AIU), Ghabaghib, Syria; 4 Unit of Pediatric Endocrinology and Metabolism, Children’s Hospital, Damascus, Syria; 5 Faculty of Medicine, Damascus University, Damascus, Syria

**Keywords:** Congenital adrenal hyperplasia, Syria, Case report

## Abstract

**Background:**

One of the most common types of congenital adrenal hyperplasia is an autosomal recessive disorder with 21-hydroxylase deficiency. The classical form, defined by cortisol insufficiency, is accompanied by prenatal androgen excess causing variable masculinization degrees of external genitalia in babies with a 46, XX karyotype.

**Cases presentation:**

These five case reports highlight the management of Syrian females aged between 0 and 32 years with congenital adrenal hyperplasia. Two of the patients have been raised as males, while two had reconstructive surgery and one had hormonal therapy. Becoming mother was achieved by two patients

**Conclusion:**

The integrated treatment of females with classical congenital adrenal hyperplasia CAH, which includes appropriate surgical procedures and controlled hormonal therapy, gives these females the opportunity to live as they are, and perhaps as mothers in the future.

## Introduction

Congenital adrenal hyperplasia (CAH) with 21-hydroxylase deficiency (21-OHD) deficiency is an autosomal recessive disorder [18] caused by mutations in the *CYP21A2* gene [19], and is considered one of the most common causes of genital ambiguity [28]. CAH clinical manifestations have been classified into two forms: classical CAH, which includes two types, severe salt wasting (SW) and non-SW or simple virilizing (SV), and a nonclassical (NC) form, in which postnatal androgen excess occurs during late puberty or early adulthood [12].

Female patients having a 46, XX karyotype with classical CAH are born with variable degrees of external genital ambiguity due to excess androgen during *in utero* development [3]. In addition to ambiguous female genitalia, affected patients with SW may exhibit life-threatening salt-losing crises during the neonatal period. Accelerated growth and skeletal maturation, hirsutism, oligomenorrhea, and infertility are faced later in life if patients were not treated or are affected by NC [25]. Appropriate treatment, regular follow-ups, and patient compliance are crucial factors for successful pregnancies. However, psychosocial factors or sexual orientation and the will to bear children influence the fertility rate [17].

In Syria, in cases of virilized external genitalia, parents used to consider these females newborns as males, especially in cases of severe virilization, until proven otherwise.

Almost all females with SW-CAH and more than 50% of those with SV-CAH had undergone at least one genital surgery on the clitoris and/or the vagina early in their childhood [23]. Early surgical treatment, rather than delayed or staged approaches for 46, XX CAH patients with specific degrees of genital virilization, has been included in guidelines for the Development of Comprehensive Care Centers for Congenital Adrenal Hyperplasia [4].

The role of the parents in sex assignment becomes crucial in all aspects of the decision-making process and all possible therapeutic options for the intersex child, particularly early versus delayed surgery [8].

Here we report the cases of five CAH females, two of them have been raised as males, two had reconstructive surgery, and one had hormonal therapy.

## Materials and methods

### Patients

The patients were being treated in the Children University Hospital, Al-Assad University Hospital, or in private endocrinology clinics between 2017 and 2020 in Damascus. Informed consent forms were signed by the patient or her guardian. This study was approved by the Damascus University research ethics committee (HRECPHARMDU Resolution No. 2). To establish the clinical diagnosis of 21-OHD, we collected the data from the medical records of each patient, including measurements of serum electrolytes, 17-hydroxy progesterone (17-OHP), glucose, bone age determination, and abdominal and pelvic ultrasonography. Cytogenetic evaluation was performed for sex determination.

### Sample preparation

Peripheral blood samples were drawn from patients on ethylenediaminetetraacetic acid (EDTA) and frozen at −20 °C. We preformed DNA extraction using the QIAamp DNA Mini Kit (Qiagen, Germany) and measured DNA concentration using NanoVue (Biochom, UK). The extracted DNA sample was kept at  4 °C and thereafter used for mutation analysis as reported by [26]. Samples used for karyotype testes were heparinized peripheral blood and the Giemsa staining protocol was applied [15].

### Case 1

**Fig. 1:**
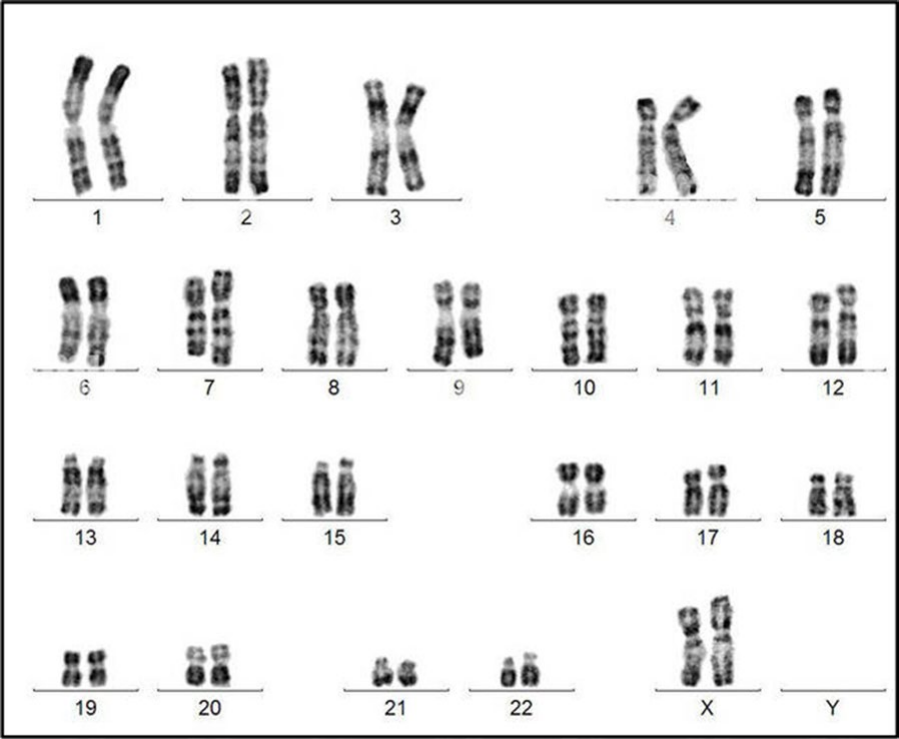
Karyotype results in case 1

In 2011, a first baby (46, XX) (Fig. 1) was born from a marriage between cousins. The baby had male-appearing external genitalia and untraceable testes. The abdominal ultrasound revealed the presence of a uterus and ovaries. Blood analysis shown high level of 17-OHP and normal levels of Na and K (Table 1). This baby girl had SV-CAH, due to the hetero compound mutations V281L and Q318X [11]. She was treated with hydrocortisone (20 mg/day).

**Table 1:** Patients results at diagnosis

Case	Na (135–145 mEq/L)	K (3.5–5.0 mEq/L)	ACTH (< 46 ng/L)	17-OHP (< 400 ng/dL)	LH (< 0.5 IU/L)	FSH (< 22 IU/L)	TSH (0.76–5.91 mIU/L)
1	136	4.6	320	40	0.3	0.5	3.2
2	120	8	920	35	0.12	0.1	4.6
3	124	7	690	45	1	0.3	1.2
4	129	6.5	620	41	0.5	0.3	4.8
5	137	4.2	452	25	2	0.2	2.8

17 OHP: 17-alpha-hydroxy progesterone; TSH: thyroid stimulating hormone; LH: luteinizing hormone; FSH: follicle stimulating hormone; ACTH: adrenocorticotropic releasing hormone; (…): reference ranges

Because the baby’s aunts had been previously diagnosed with classical CAH with ambiguous genitalia, her parents made the decision of eradicating the uterus and ovaries, and preserving the masculine appearance in the absence of gonads, regardless of the endocrinologists’ advice. This female was raised as a male but he is infertile, and the outcome of genital surgery was negative due to gender reassignment as a boy. She had not got menarche.

However, this infant has a sister who was born with SV-CAH and ambiguous genitalia, but who underwent reconstructive surgeries and was treated as a girl.

### Case 2

**Fig. 2:**
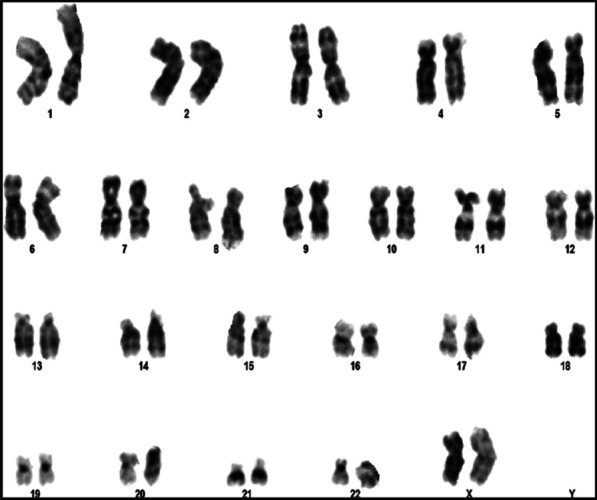
Karyotype results in case 2

The first child of 5 years old was born to an unrelated couple in 1998. Despite the presence of ambiguous genitalia (Tunner V), the abdominal echography revealed adrenal hypertrophy and a vagina. High levels of 17-OH and potassium were detected, in addition to low levels of sodium (Table 1). This baby girl (46, XX) (Fig. 2), had SW-CAH due to the inheritance of the homozygote mutation Q318X [11]. She was treated by hydrocortisone (30 mg/day) and fludrocortisone (0.1 mg/day). Only one reconstructive surgery was performed on her at the age of two, but the parents chose to treat her as a boy. Puberty had begun at age of 9 years, but it was disabled to increase the stature using hormonal therapy. Facial and pubic hirsutism appeared: she had a male look with beard and mustache, and she went to serve in the military. This case treatment had a negative outcome, and she had not got menarche due to hormonal therapy.

She had a sister with SW-CAH, who underwent reconstructive surgeries and was treated as a girl.

### Case 3

**Fig. 3:**
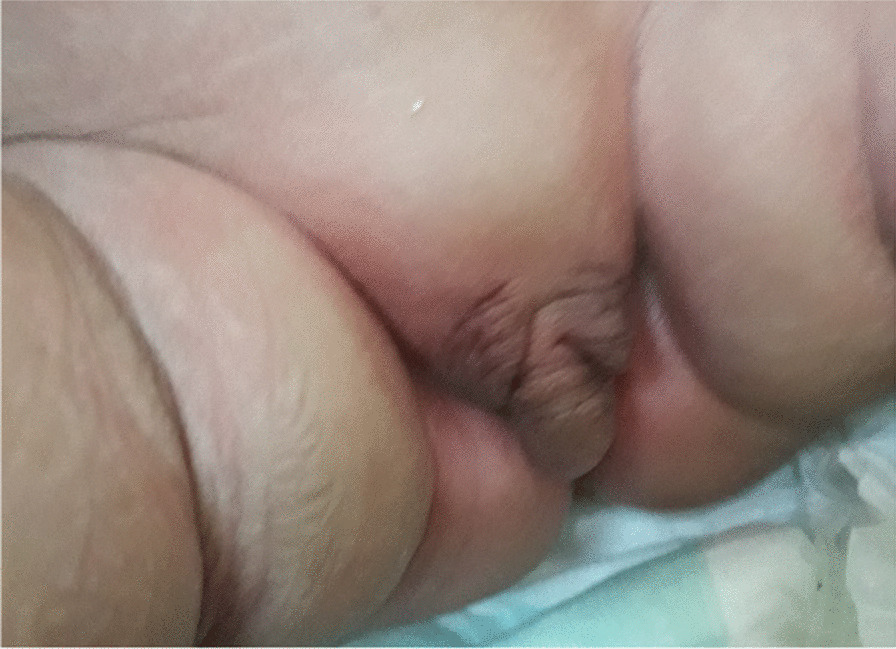
Ambiguous genitalia in case 3

**Fig. 4:**
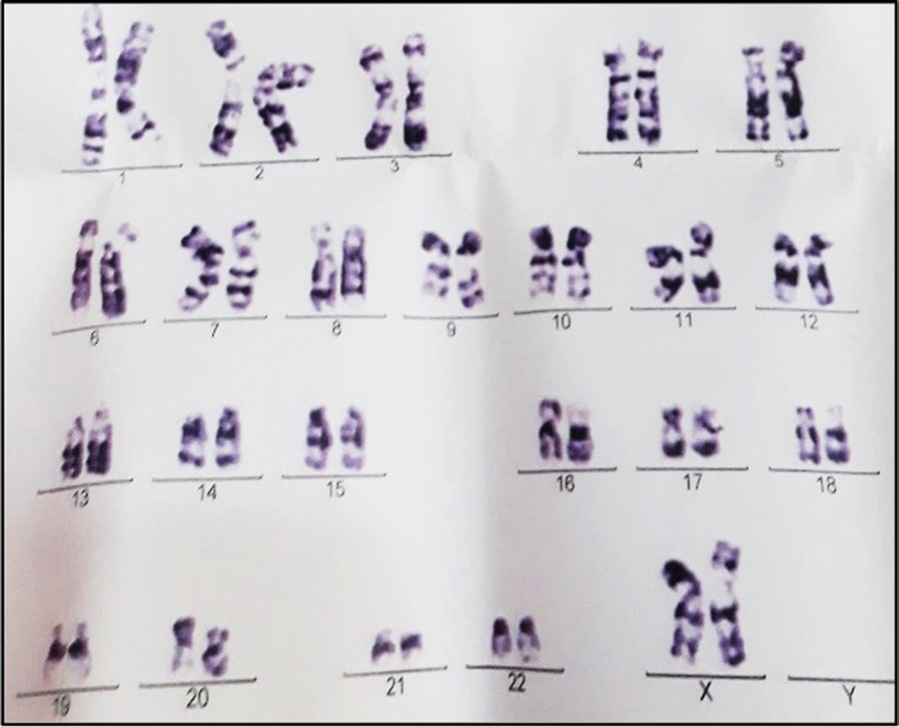
Karyotype results in case 3

A baby was born in 2017 to a couple of cousins, she was their third child. At birth, ambiguous genitalia were present (Fig. 3), karyotyping revealed 46, XX (Fig. 4), and echography detected a uterus and ovaries. She had SW-CAH due to the inheritance of two heterozygote mutations I2G and V281L, in addition to the homozygote mutation I172N [11]. Hyponatremia and hyperkalemia were detected (Table 1). Hydrocortisone (20 mg/day) and fludrocortisone (0.1 mg/day) were used as treatment, and the baby girl underwent several reconstructive surgeries to correct her external genitalia. This is a positive outcome as natal gender was preserved.

### Case 4

**Fig. 5:**
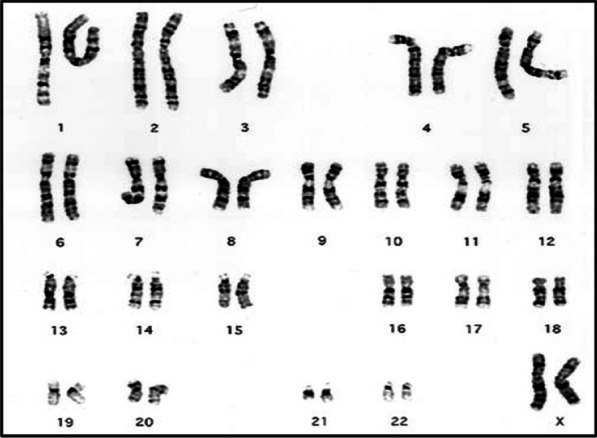
Karyotype results in case 4

A female was born in 2002 to a couple of cousins. Ambiguous genitalia were present at birth. Karyotyping revealed 46, XX (Fig. 5), echography detected a uterus and ovaries, and 17-OHP was elevated (Table 1). She had SW-CAH, but mutations were not explored. She was treated with hydrocortisone (10 mg/day) and fludrocortisone (0.1 mg/day), and underwent reconstructive surgery at the age of 18 months. At the age of 15 years, she reached puberty, and she was followed-up well by the endocrinologist. She got married in 2020 and had a baby. A positive outcome.

### Case 5

A female was born in 1990 to a couple of cousins. She was their first child out of four children (three females and one male).

At the age of 4 years, she was diagnosed with SV-CAH owing to the appearance of hirsutism in the genital areas, slight enlargement in the clitoris, and the detection of high levels of 17-OHP (Table 1). She got menarche at 14 years old, but a menstrual disorder appeared later. She had compound heterozygous of I2G and R356W mutations [10]. She underwent treatment with hydrocortisone (20 mg/day) and was followed-up by an endocrinologist for the treatment of her menstrual disorder. She got married in 2014 and had twin daughters.

## Discussion

One of the genetic diseases prevalent in Syria is CAH caused by a deficiency of the enzyme 21-OH. The most important factor affecting its heredity is consanguineous marriage [11]. A relatively high consanguinity rate of 30–39% is reported among the Syrian population [24].

The percentage of consanguineous marriage in CAH studies conducted in Iraq was 82% [2], 63% in Jordan [7], 40% in Lebanon [9], 65.5% in Egypt [13], 28.8% in Turkey [5], and 57.1% in Iran [14]. It is important to avoid this regional social habit that helps spread the disease.

The differences in health care provision and treatment regimens affect the quality of life for patients with CAH. Challenges allegedly vary according to sex, but all patients are subject to the emotional stress of living with a chronic disease, and many have weight and height disturbances and are at risk of infertility [22].

CAH Patients should be followed-up by a multidisciplinary team including a gynecologist, endocrinologist, pediatrician, and a psychologist [6].

Female patients (46, XX) with CAH are born with variable degrees of external genital ambiguity due to excess androgen during *in utero* development. Their internal reproductive anatomy includes a normal uterus, fallopian tubes, and ovaries with a potential for fertility. The ambiguous genitalia, and the often-related uncertainty regarding the gender assignment, can represent psychosocial trauma for the family, and carries a risk of social stigma for the patient [3].

The study we conducted to identify the eight most common mutations in patients with classical CAH showed that the rate of female genital ambiguity is high in classical CAH (27/38 CAH female) [11]. Upon reviewing the medical archives of a group of affected females with CAH, we noticed the role of the parents in making the decision to determine the gender of the newborn, and therefore the type of surgical procedure that is appropriate for the chosen gender. In the majority of cases, the chosen gender is compatible with the 46, XX karyotype.

In cases 1 and 2 in the present study, the masculine gender was selected by parents according to regional and cultural traditions of having a male that can be relied upon, despite the genetic and endocrinologist counseling. A similar unethical practice was also found in India [21], Pakistan [16], Algeria [20], and the Kingdom of Saudi Arabia [1]. The child has the right to develop according to their natal sex.

Reconstructive surgery, as in cases 3 and 4, could offer psychological relief, by resolving the sexual ambiguity of the genitalia, and may facilitate sexual intercourse, although it may enhance the feeling of being different [23]. Psychological support provides the CAH female patient and those around her with the best solution to adapt to this health condition, which she inherited from her parents. Having the female reproductive system that is proportional to the genetic pattern favors the idea of dealing with the newborn as a girl, and removes the first and main problem in the parents’ behavior, and encourages them to support her morally.

When the endocrine aspects of fertility are normalized, a normal pregnancy rate could result, as in cases 4 and 5. Suppression of testosterone hyper secretion, which induces anovulation, is relatively simple to achieve. The consequence of genital surgery is a key factor for the fertility outcome as vaginal function and sexual activity are closely related [27]. Recently, it was found that the fertility rate increases by 90% in women with classical CAH when they follow the stages of integrated treatment for CAH [17].

## Conclusion

The integrated treatment of females with classical CAH, which includes psychological support in addition to the use of appropriate hormones, appropriate surgical procedures to correct the external genitalia of an ambiguous appearance into a female appearance in the presence of a female karyotype, and the corresponding reproductive system, gives these females the opportunity to live as they are and perhaps as mothers in the future. Patients with CAH should be followed by a team including a gynecologist, endocrinologist, pediatrician, and a psychologist.

## Data Availability

All the information about patients is in their medical records with the clinicians participating in this article.

## Abbreviations

CAH: Congenital adrenal hyperplasia
C: Classical
NC: Non-classical
SW: Salt wasting
SV: Simple virilizing
17-OHP: 17-hydroxy progesterone
